# Biflavonoids Isolated from *Selaginella tamariscina* and Their Anti-Inflammatory Activities via ERK 1/2 Signaling

**DOI:** 10.3390/molecules23040926

**Published:** 2018-04-17

**Authors:** Sun-Yup Shim, Seul-gi Lee, Mina Lee

**Affiliations:** 1 College of Pharmacy, Sunchon National University, 255 Jungangno, Suncheon-si 57922, Jeonnam, Korea; shimsy@sunchon.ac.kr (S.-Y.S.); thelsg@sunchon.ac.kr (S.-g.L.); 2 Research Institute of Life and Pharmaceutical Sciences, 255 Jungangno, Suncheon-si 57922, Jeonnam, Korea

**Keywords:** *Selaginella tamariscina*, hinokiflavone, 7′-*O*-methyl hinokiflavone, RAW 264.7 cells, HT-29 cells

## Abstract

*Selaginella tamariscina* (*S. tamariscina*) (Beauv.) Spring (Selaginellaceae) has been used in oriental medicine for the treatment of dysmenorrhea, chronic hepatitis, hyperglycemia, amenorrhea, hematuria, prolapse of the anus and metrorrhagia. In the present study, we isolated two strong anti-inflammatory compounds, the biflavonoids hinokiflavone (H) and 7′-*O*-methyl hinokiflavone (mH), from *S. tamariscina* and examined their anti-inflammatory activities in lipopolysaccharide (LPS)-mediated murine macrophages (RAW 264.7) and colon epithelial cells (HT-29). H and mH suppressed the production of the inflammatory mediators nitric oxide (NO), interleukin (IL)-6, IL-8, and tumor-necrosis factor (TNF)-α, which are most highly activated in inflammatory bowel disease (IBD). In addition, Western blot analysis revealed that H and mH suppressed the LPS-induced expression of inducible nitric oxide synthase (iNOS) and cyclooxygenase (COX)-2, and the activation of nuclear factor-κB (NF-κB) and extracellular regulated kinases (ERK) 1/2. These results suggest that H and mH are compounds having potent anti-inflammatory effects that could be used to treat such diseases as IBD.

## 1. Introduction

Inflammation is part of our innate immunity, and is an important host defense response to injury, tissue ischemia, and autoimmune responses or pathogens, and consists of a complicated set of events regulated by various chemical mediators released by both resident and infiltrating cells [1,2]. Inflammatory bowel diseases (IBDs)—principally, ulcerative colitis and Crohn′s disease—are characterized by chronic inflammatory disease in the gastrointestinal tract due to the transmural infiltration of immune cells, leading to the disruption of the mucosa and, ultimately, to ulceration. These diseases are caused by various environmental and genetic factors [3]. Immune cells involving macrophages, mast cells, and lymphocytes play an important role as effector cells in IBD. Activated immune cells are associated with elevated levels of various inflammatory mediators involving interleukin (IL)-1β, IL-6, IL-8, tumor necrosis factor (TNF)-α, nitric oxide (NO), reactive oxygen species (ROS), inducible nitric oxide synthase (iNOS), and cyclooxygenase (COX)-2 [4,5,6,7].

Nuclear factor-kappa B (NF-κB) is a proinflammatory transcription factor and is primarily regulated by inflammatory mediators which have important roles in the pathogenesis of inflammatory diseases, such as IBDs, and are produced in response to lipopolysaccharide (LPS). Mitogen-activated protein kinases (MAPKs) such as extracellular regulated kinase (ERK), p38, and c-Jun-N-terminal kinase (JNK) in signaling plays an important role in NF-κB activation, and the downregulation of mucosal NF-κB signaling may prevent IBD [8,9,10,11,12].

*Selaginella tamariscina* (*S. tamariscina*) (Beauv.) Spring (Selaginellaceae) has been used in oriental medicine for the treatment of dysmenorrhea, chronic hepatitis, hyperglycemia, amenorrhea, hematuria, prolapse of the anus, and metrorrhagia. Pharmacological studies on *S. tamariscina* have reported its anti-inflammatory, antibacterial, anti-hypertensive and anti-hyperglycemic activities [13,14]. The investigation of the phytochemical constituents of *S. tamariscina* revealed it to be an abundant source of biflavonoids (e.g., amentoflavone, hinokiflavone, isocryptomerin, sumaflavone, and robustaflavone). The biflavonoids isolated from *S. tamariscina* are known to display a variety of biological activities involving anti-inflammatory, anti-allergic, antitumor, antioxidant, antidiabetic, antiviral, and anticancer activities, and osteogenesis [15,16,17,18,19]. It was reported that amentoflavone and sumaflavone isolated from *S. tamariscina* have anti-inflammatory activities [20,21]. However, the anti-inflammatory activities of hinokiflavone (H) and methyl hinokiflavone (mH) isolated from *S. tamariscina* have not yet been investigated. In the present study, the potent anti-inflammatory molecular action of the biflavonoids H and mH, isolated from *S. tamariscina*, was investigated in LPS-induced murine macrophage and colon epithelial cells.

## 2. Results

### 2.1. Preliminary Determination of the Effects on NO Production

**Figure 1:**
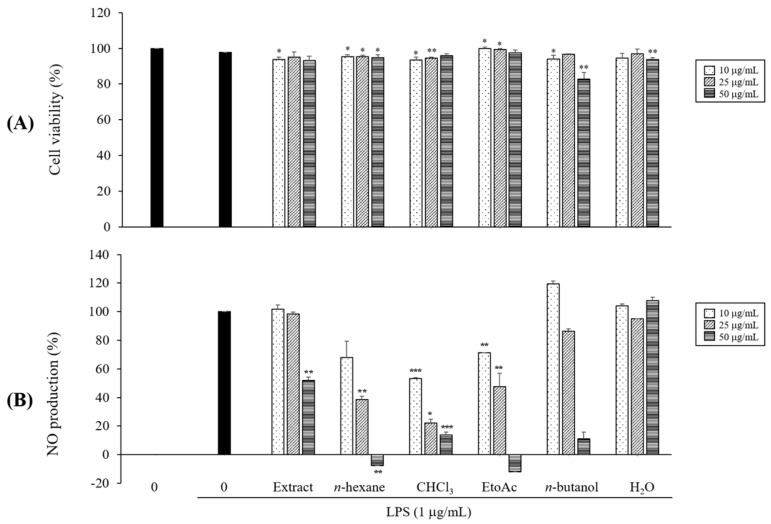
Cell viability (**A**) and nitric oxide (NO) production (**B**) of total extract and fractions from *Selaginella tamariscina.* RAW 264.7 cells were cultured in the presence of total extract and each fraction for 1 h and stimulated with lipopolysaccharide (LPS) (1 μg/mL) for 24 h under serum-free conditions. Cell viabilities and NO production were determined using cell counting kit (CCK)-8 assay and Griess reagent, respectively. Nitrite concentrations of nontreated and LPS-treated controls were 4.63 ± 1.48 μM and 30.35 ± 0.12 μM, respectively. Each determination was made in triplicate. Data are presented as means ± SD. * *p* < 0.05, ** *p* < 0.01, *** *p* < 0.001 vs. LPS-treated group.

While searching for anti-inflammatory natural products targeting the treatment of IBD, we found that the methanolic extract of *S. tamariscina* has an inhibitory effect on NO production in LPS-induced macrophages. This methanolic extract was then suspended in distilled water (DW) and successively partitioned with *n*-hexane, CHCl_3_, EtOAc, and *n*-butanol, and each fraction had no cytotoxicity at a range of ~50 μM (Figure 1A). As a result of the evaluation of the anti-inflammatory activities of each fraction, the EtOAc fraction showed the most significant inhibitory effect on LPS-induced NO production (Figure 1B).

### 2.2. Isolation and Structural Determination of Biflavonoids from the EtOAc Fraction

**Figure 2:**
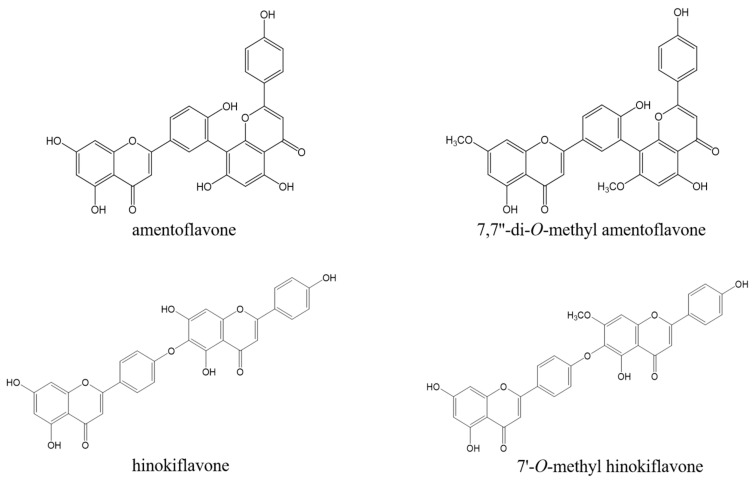
Chemical structure of four compounds isolated from *S. tamariscina.*

We further isolated the EtOAc fraction using column chromatography and obtained four compounds. These compounds were identified as amentoflavone (**1**), 7,7″-*O*-dimethyl-amentoflavone (**2**), hinokiflavone (**3**), and 7″-*O*-methyl hinokiflavone (**4**) by comparison of the spectral data, including 1D and 2D NMR, with those reported in the literature (Figure 2) [22,23,24].

Amentoflavone (**1**). Yellow powder; C_30_H_18_O_10_; ESIMS (positive mode): *m*/*z* 538.46 [M]^+^; ^1^H-NMR (400 MHz, DMSO-*d*_6_): δ_H_ 8.02 (1H, dd, *J* = 2.3, 8.7 Hz, H-6′), 8.00 (1H, d, *J* = 2.3 Hz, H-2′), 7.58 (2H, d, *J* = 9.2 Hz, H-2′′′, 6′′′), 7.15 (1H, d, *J* = 9.6 Hz, H-5′), 6.85 (H, s, H-3), 6.81 (1H, s, H-3′′), 6.72 (1H, d, *J* = 9.2 Hz, H-3′′′, 5′′′), 6.39 (1H, d, *J* = 1.8 Hz, H-8), 6.40 (1H, s, H-6′′), 6.19 (1H, d, *J* = 2.3 Hz, H-6); ^13^C-NMR (100 MHz, DMSO-*d*_6_): δ_C_ 183.1 (C-4), 182.7 (C-4′′), 165.1 (C-2′′), 164.8 (C-2), 164.7 (C-7′′), 162.8 (C-7), 162.4 (C-4′), 162.0 (C-5′′), 161.5 (C-5), 160.5 (C-4′′′), 158.3 (C-9′′), 155.5 (C-9), 132.4 (C-2′′′), 129.2 (C-2′, 6′), 128.8 (C-6′′′), 122.4 (C-1′), 122.0 (C-3′′′), 121.0(C-1′′′), 117.1 (C-5′′), 116.7 (C-3′, 5′), 104.9 (C-8), 104.7 (C-10), 104.0 (C-3), 103.6 (C-3′′), 99.8 (C-6′′), 99.6 (C-6), 95.0 (C-8′′).

7,7′′-*O*-dimethyl-amentoflavone (**2**). Yellow powder; C_32_H_22_O_10_; ESIMS (negative mode): *m/z* 565.12 [M − H]^−^; ^1^H-NMR (400 MHz, DMSO-*d*_6_): δ_H_ 8.02 (1H, dd, *J* = 2.3, 8.7 Hz H-6′), 8.00 (1H, d, *J* = 2.3 Hz, H-2′), 7.68 (1H, dd, *J* = 9.2 Hz, H-2′′′, 6′′′), 7.15 (1H, d, *J* = 8.7 Hz, H-5′), 6.94 (1H, s ,H-3), 6.92 (2H, d, *J* = 9.2 Hz, H-3′′′, H-5′′′), 6.82 (3H, s, H-3′′), 6.68(3H, s, H-6′′), 6.44 (H, d, *J* = 1.8 Hz, H-8), 6.16 (H, d, *J* = 1.8 Hz, H-6), 3.84 (3H, s, OCH_3_-7), 3.77 (3H, s, OCH_3_-7′′); ^13^C-NMR (100 MHz, DMSO-*d*_6_): δ_C_ 182.5 (C-4), 181.8 (C-4′′), 169.1 (C-7′), 164.5 (C-7′′), 163.7 (C-2, C-2′′), 162.4 (C-8), 161.5 (C-4′, C-5′′), 157.5 (C-4′′′), 153.7 (C-9′′), 135.7 (C-2′), 128.2 (C-2′′′, 6′′′), 125.9 (C-6′), 122.9 (C-1′), 119.6 (C-1′′′), 117.4 (C-3′), 116.3 (C-5′), 114.6 (C-3′′′, 5′′′), 105.1 (C-10), 103.7 (C-10′′), 103.3 (C-3, 3′′), 95.6 (C-8′′), 94.2 (C-6,6′′), 91.3 (C-8), 57.0 (OCH_3_-7′′), 56.1 (OCH_3_-7).

Hinokiflavone (**3**). White powder; C_30_H_18_O_10_; ESIMS (negative mode): *m/z* 537.08 [M-H]^-^; ^1^H-NMR (400 MHz, DMSO-*d*_6_): δ_H_ 8.02 (2H, d, *J* = 8.7 Hz, H-2′, 6′), 7.97 (2H, d, *J* = 8.7 Hz, H-2′′′, 6′′′), 7.04 (2H, d, *J* = 8.7 Hz, H-3′, 5′), 6.94 (2H, d, *J* = 8.7 Hz, H-3′′′, 5′′′), 6.86 (1H, s, H-3), 6.85 (1H, s, H-3′′), 6.71 (1H, s, H-8′′), 6.49 (1H, d, *J* = 1.8 Hz, H-8), 6.20 (1H, d, *J* = 1.8 Hz, H-6); ^13^C-NMR (100 MHz, DMSO-*d*_6_): δ_C_ 182.0 (C-4′′), 181.8 (C-4), 164.3 (C-7), 164.1 (C-2′′), 163.2 (C-2), 161.4 (C-5), 161.3 (C-4′′′), 160.7 (C-4′), 157.5 (C-9), 157.3 (C-9′′), 153.8 (C-7′′), 153.1 (C-5′′), 128.6 (C-2′′′, 6′′′), 128.3 (C-2′, 6′), 124.7 (C-6′′), 124.2 (C-1′), 121.1 (C-1′′′), 116.0 (C-3′′′, 5′′′), 115.3 (C-3′, 5′), 103.9 (C-10, 10′′), 103.8 (C-3), 102.5 (C-3′′), 98.9 (C-6), 94.6 (C-8′′), 94.0 (C-8).

7”-*O*-Methyl hinokiflavone (**4**). Yellow powder; C_31_H_20_O_10_; ESIMS (negative mode): *m/z* 552.08 [M − H]^−^; ^1^H-NMR (400 MHz, DMSO-*d*_6_): δ_H_ 8.00 (4H, d, *J* = 8.7 Hz, H-2′,6′, 2′′,6′′), 7.09 (1H, s, H-3′′), 6.99 (2H, d, *J* = 8.7 Hz, H-3′, 5′), 6.89 (2H, d, *J* = 8.7 Hz, H-3′′, 5′′), 6.92 (1H, s, H-3) 6.85 (1H, s, H-8), 6.48 (1H, d, *J* = 1.8 Hz, H-8′′), 6.20 (1H, d, *J* = 1.8 Hz, H-6), 3.89 (3H, s, OCH_3_-7′′); ^13^C-NMR (100 MHz, DMSO-*d*_6_): δ_C_ 182.7 (C-4′′), 182.3 (C-4), 164.9 (C-7), 164.8 (C-2′′), 163.6 (C-2), 162 (C-5), 162 (C-4′′′), 161.1 (C-4′), 159 (C-9), 157.9 (C-9′′), 154.6 (C-5′′), 152.9 (C-7′′), 129.2 (C-2′′′), 129.2 (C-6′′′), 128.9 (C-2′′, 6′′), 125.3 (C-1′), 124.9 (C-1′′′), 116.5 (C-3′′′, 5′′′), 115.7 (C-3′, 5′), 105.7 (C-10), 104.5 (C-10′′), 104.3 (C-3), 103.4 (C-3′′), 99.4 (C-6), 94.5(C-8), 92.6 (C-8′′), 57.3 (OCH_3_-7′′).

Among the four compounds with an inhibitory action on NO production, the anti-inflammatory effects of amentoflavone and 7,7′′-*O*-dimethyl-amentoflavone were previously reported [25,26,27]. The anti-inflammatory molecular action of H and mH isolated from *S. tamariscina* has not yet been examined. In the present study, we focused on the anti-inflammatory molecular activities of biflavonoids isolated form *S. tamariscina* in LPS-induced murine macrophage and colon epithelial cells leading to the protection of IBD.

### 2.3. Effects on Cytotoxicity

**Figure 3:**
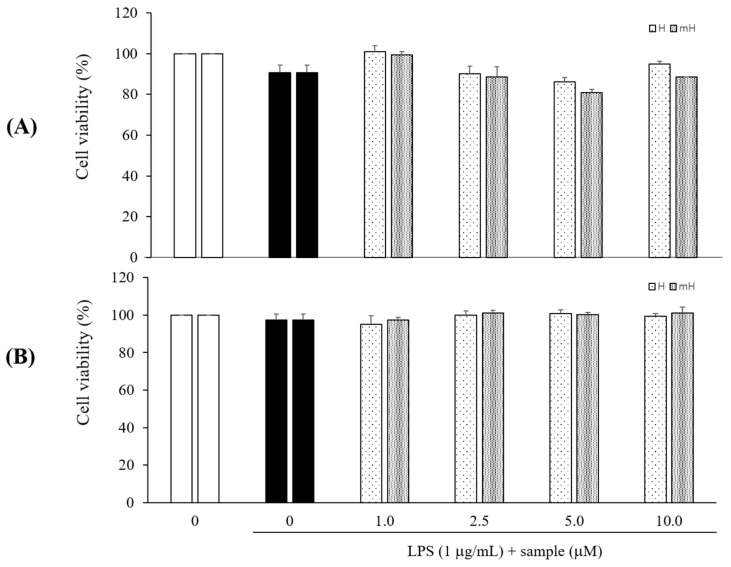
Effects of H and mH on cell viability in LPS-induced RAW 264.7 (**A**) and HT-29 cells (**B**). Cells were cultured in the presence of H and mH for 1 h and stimulated with LPS (1 μg/mL) for 24 h under serum-free conditions. Cell viabilities were determined using CCK-8 assay. Each determination was made in triplicate. Data are presented as means ± SD.

LPS signals are potent stimuli known to induce inflammatory mediators, while macrophages and colon epithelial cells are effector cells in IBD and are the major cellular targets for LPS [28]. The cytotoxicity of H and mH isolated from *S. tamariscina* was assessed by quantitating the cell viabilities in the presence of these flavonoids. The cells were pretreated with H and mH, stimulated with LPS for 24 h, and then analyzed for cytotoxicity using CCK-8. These flavonoids exhibited no toxic effects on the cells over the concentration range from 1 to 10 μM in comparison to control cells that received no treatment in LPS-induced RAW 264.7 cells (Figure 3A) and HT-29 cells (Figure 3B).

The subsequent experiments were performed with H and mH at concentrations ranging from 1 to 10 μM.

### 2.4. Effects on NO Production

**Figure 4:**
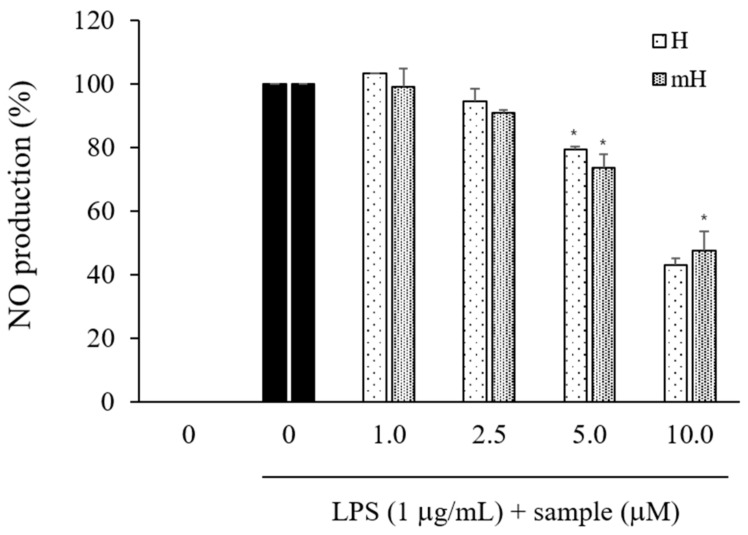
Effects of H and mH on NO production in LPS-induced RAW 264.7. Cells were cultured in the presence of H and mH for 1 h and stimulated with LPS (1 μg/mL) for 24 h under serum-free conditions. NO production was determined using Griess reagent. Nitrite concentrations of nontreated and LPS-treated controls were 4.63 ± 1.48 μM and 30.35 ± 0.12 μM, respectively. Each determination was made in triplicate. Data are presented as means ± SD. * *p* < 0.05 vs. LPS-treated group.

To investigate the effects of *S. tamariscina* on inflammation, the inhibitory effects of its flavonoids on the production of NO were determined in culture media consisting of LPS-stimulated RAW 264.7 cells via the Griess reaction. The LPS-induced NO production was observed to decrease by treatment with H and mH in a concentration-dependent manner (Figure 4).

### 2.5. Effects on iNOS and COX-2 Expression

**Figure 5:**
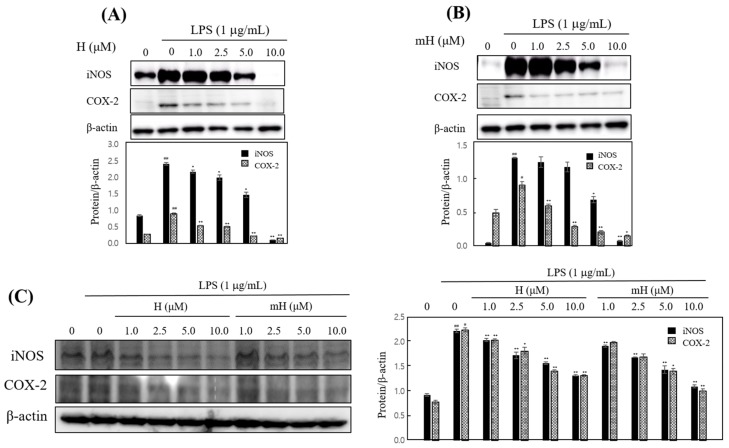
Effects of H and mH on inducible NO synthase (iNOS) and cyclooxygenase (COX)-2 expression. Cells were cultured in the presence of H and mH for 1 h and stimulated with LPS (1 μg/mL) for 24 h under serum-free conditions. The expression of iNOS, COX-2, and β-actin in LPS-induced RAW 264.7 (**A**,**B**) and HT-29 cells (**C**) was detected by Western blot analysis. The relative density was calculated as the ratio of each protein expression to β-actin. ^#^ *p* < 0.05, ^##^ *p* < 0.01 vs. control; * *p* < 0.05, ** *p* < 0.01 vs. LPS-treated group.

To confirm these anti-inflammatory effects, we investigated the inhibitory effect of H and mH against the overexpression of iNOS and COX-2. H and mH inhibited the expression of iNOS and COX-2 in LPS-induced RAW 264.7 cells (Figure 5A,B) and HT-29 cells (Figure 5C) in a concentration-dependent manner. These results suggest that the H- and mH-mediated inhibition of inflammatory mediator production is associated with the negatively regulated transcription of iNOS and COX-2.

### 2.6. Effects on Proinflammatory Cytokines Production

**Figure 6:**
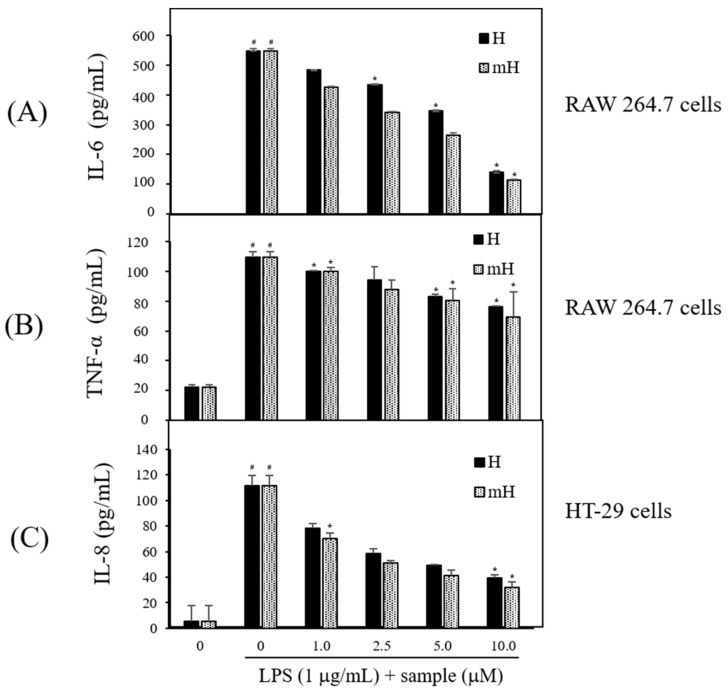
Effects of H and mH on production of proinflammatory cytokines. Cells were pretreated with H and mH for 1 h and then stimulated with LPS (1 μg/mL) for 24 h. Levels of interleukin (IL)-6 (**A**); tumor-necrosis factor (TNF)-α (**B**); and IL-8 (**C**) were determined by ELISA in culture media of RAW 264.7 (IL-6, TNF-α) and HT-29 cells (IL-8), respectively. Each determination was made in triplicate. Data are presented as means ± SD. ^#^ *p* < 0.05 vs. control; * *p* < 0.05 vs. LPS-treated group.

Proinflammatory cytokines are secreted at an early stage of the inflammatory reaction and are recognized as a key marker of inflammation [11,12]. To assess the potential anti-inflammatory effect of the biflavonoids, H and mH, on the inflammatory signaling of LPS-induced macrophage and epithelial cells, the LPS-stimulated production of inflammatory cytokines was measured by ELISA. Treatment with H and mH (1~10 μM) concentration-dependently inhibited proinflammatory cytokines IL-6 (Figure 6A) and TNF-α (Figure 6B) in LPS-induced RAW 264.7 cells. Moreover, H and mH inhibited IL-8 production in LPS-induced HT-29 cells (Figure 6C). These results show that the inhibition rate of TNF-α was lower than that of inflammatory cytokines such as IL-6 and IL-8.

### 2.7. Effects on NF-κB Expression

**Figure 7:**
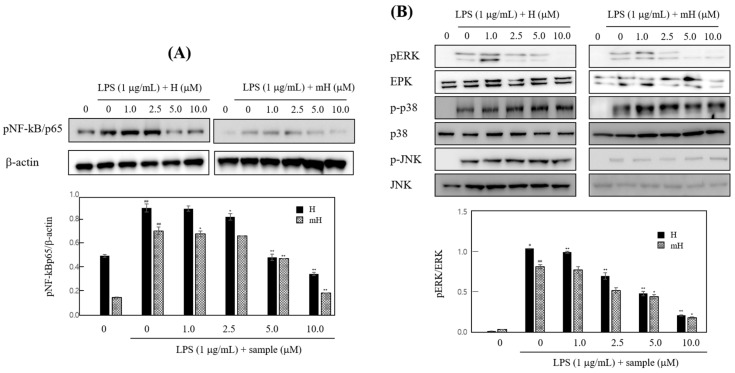
Effects of H and mH on nuclear factor-κB (NF-κB) (**A**) and mitogen-activated protein kinase (MAPK) (**B**) activation. Cells were cultured in the presence of H and mH for 24 h and then stimulated with LPS (1 μg/mL) for 15 min under serum-free condition. The expression of NF-κB and MAPK was detected by Western blot analysis. The relative density of phosphorylated NF-κB (pNF-κB) and phosphorylated extracellular regulated kinase (pERK) was calculated as the ratio of each protein expression to β-actin and ERK, respectively. ^#^ *p* < 0.05, ^##^ *p* < 0.01 vs. control; * *p* < 0.05, ** *p* < 0.01 vs. LPS-treated group.

To determine transcriptional regulation of H and mH, we investigated the activities of H and mH on the transcriptional activation of NF-κB in LPS-stimulated RAW 264.7 cells. As shown in Figure 7A, H and mH suppressed LPS-induced NK-κB activation in a concentration-dependent manner. Mitogen-activated protein kinases (MAPK) pathways are considered a major mechanism in inflammatory reactions and are involved in the regulation of NF-κB signaling [29,30,31]. We examined the effect of H and mH on the phosphorylation of three MAPKs—ERK 1/2, p38, and JNK—in LPS-induced murine macrophages by Western blot analysis. H and mH inhibited the phosphorylation of ERK in a concentration-dependent manner (Figure 7B). Moreover, H and mH did not affect phosphorylation of JNK and p38 (Figure 7B).

## 3. Discussion

Numerous natural plants have been used as traditionally prescribed herbal plants for treatment of various diseases. *S. tamariscina* is a representative of such an ethnopharmacologically used plant in East Asia [13]. As part of a search for natural therapeutic products against inflammation, this study was performed to investigate the intestinal anti-inflammatory action of the biflavonoids H and mH, isolated from *S. tamariscina*, in LPS-stimulated murine macrophages and human epithelial cells for the protection of IBD. To determine the concentration of H and mH to be used in all of the following experiments, their cytotoxic effects on the cell proliferation of RAW 264.7 and HT-29 cells were investigated, and it was found that these flavonoids exhibited no toxic effects on the cells over the concentration range from 1 to 10 μM (Figure 3). NO—which is an inflammatory regulatory mediator in the physiological and pathological processes of inflammation and is produced mainly by activated macrophages—was suppressed by H and mH (Figure 4). It has been reported that iNOS and COX-2 are necessary for the production of inflammatory mediators [4]. Our results revealed that H and mH inhibited the protein expression levels of iNOS and COX-2 in an LPS-stimulated condition (Figure 5). These results showed that H- and mH-mediated inhibition of the inflammatory mediators is likely due to the downregulation of iNOS and COX-2 activation.

MAPK plays a critical role in the regulation of cellular signaling pathways, such as cell death, apoptosis, inflammation, and allergic reactions [12,29]. Thus, we examined the effects of H and mH on the activation of ERK 1/2, p-38, and JNK 1/2, and it was found that these compounds downregulated their ERK 1/2 activation in a concentration-dependent manner (Figure 7B). Moreover, MAPK activation regulates NF-κB signaling pathways and is involved in cell death, differentiation, and inflammation [30,31].

To further examine the molecular mechanism of H and mH in inflammation, we assessed the inhibitory action of H and mH on the activation of NF-κB in LPS-stimulated RAW 264.7 cells. Western blot analysis showed that H and mH inhibit NF-kB activation (Figure 7A). These results show that suppression of MAPK plays crucial role in decreasing H- and mH-induced anti-inflammatory action, and further supports the hypothesis of downregulation of NF-κB activation via the inhibition of ERK-1 phosphorylation. Therefore, it is likely that the H- and mH-mediated suppression of LPS-induced proinflammatory cytokines in murine macrophages and intestinal epithelial cells is dependent on the downregulation of the NF-κB pathway. Moreover, NF-κB plays an important role in the production of proinflammatory cytokines involving IL-6, IL-8, and TNF-α [30,31]. We found that H and mH inhibited the LPS-induced production of these cytokines (Figure 6).

It has been reported that NF-κB activation in mucosal macrophages is produced by iNOS, IL-1β, IL-6, and TNF-α in IBD patients [31]. We determined that the induction of iNOS, TNF-α, IL-6, and IL-8 was suppressed by treatment with H and mH in LPS-induced RAW 264.7 cells and HT-29 cells. These results demonstrate that H and mH inhibited proinflammatory cytokines in LPS-induced murine macrophages and intestinal epithelial cells by inhibiting NF-κB signaling via the ERK pathway. These results suggest that the biflavonoids H and mH, isolated from *S. tamariscina*, are promising novel natural therapeutic products for the protection and treatment of gastrointestinal inflammatory disorders such as IBD.

Taken together, the results of this study indicate the first evidence that H and mH inhibit the activation of macrophage and intestinal epithelial cells by acting as inflammatory mediators and cytokines during exposure to LPS. The inhibitory activities of H and mH are linked to the downregulation of NF-κB and MAPK signaling. Further studies on the protective action of H and mH in the dextran sodium sulfate-induced colitis model would be necessary to confirm their potential therapeutic application in the treatment of IBD.

## 4. Materials and Methods

### 4.1. Plant Material

The *S. tamariscina* aerial part was purchased from the www.handsherb.co.kr website—whose headquarters is located in Cheongsong-gun, Chungcheongnam-do, Korea—during January 2015. The *S. tamariscina* plant was identified by Prof. Jong Hee Park at Pusan National University. A voucher specimen (SCNUP 10A) was deposited at the laboratory of Pharmacognosy, College of Pharmacy, Sunchon National University, Suncheon-si, Jeollanam-do, Korea.

### 4.2. Extraction and Isolation

The dried aerial part (3 kg) of *S. tamariscina* was extracted three times with 80% methanol for 3 h using sonication. The extracted solution was filtered and dried to give the methanol extract (364.6 g). The extract was suspended in H_2_O and partitioned in a regular sequence with *n*-hexane, CHCl_3_, EtOAc, and *n*-butanol to obtain 64.4 g, 37.0 g, 31.7 g, and 50 g of residue, respectively. Among these fractions, the *n*-hexane, CHCl_3_, and EtOAc fractions showed a significant inhibitory effect on NO production in RAW 264.7 cells. The EtOAc fraction was used for further isolation work. The EtOAc fraction was used for VLC using a gradient solvent system (CHCl_3_ → CH_3_OH) to obtain 10 subfractions (E1~E10). E4 was subjected to MPLC (RP C_18_; H_2_O–CH_3_CN, 95:5 → CH_3_OH; 15 mL/min) to afford 61 subfractions (E4-1~E4-61). Compound **1** (17.8 mg) was isolated from E4-8. E3 was separated by VLC (silica gel; CHCl_3_–CH_3_OH–H_2_O, 400:4:1 → CH_3_OH) to yield 12 subfractions (E3-1~E3-12). E3-2 was subjected to MPLC (RP C_18_; H_2_O–CH_3_CN 95:5 → CH_3_OH; 15 mL/min) to obtain 61 subfractions (E3-2-1~E3-2-61). Compound **2** (9.1 mg) was obtained by recrystallization from E3-2-40. E6 was separated by MPLC (RP C_18_; H_2_O–CH_3_CN, 95:5 → CH_3_OH; 15 mL/min) to yield 17 subfractions (E6-1~E6-17). E6-12 and E6-16 were subjected to MPLC (RP C_18_; H_2_O–CH_3_CN, 95:5 → CH_3_OH; 15 mL/min) to obtain 61 subfractions referred to as E6-12-1~E6-12-61 and E6-16-1~E6-16-61, respectively. Compounds **3** (9.5 mg) and **4** (9.0 mg) were obtained from E6-12-2 and E6-16-14, respectively.

### 4.3. Cell Culture and Cytotoxicity Determination

The murine macrophage, RAW 264.7, human colonic epithelial cells, and HT-29 cells were obtained from the Korean Cell lines bank. The cells were maintained in Dulbecco’s modified Eagle’s medium (DMEM) supplemented with 10% heat-inactivated FBS, and Penicillin and Streptomycin Solution (HyClone, Logan, UT, USA), and cultured at 37 °C in a humidified atmosphere with 5% CO_2_. The cells were cultured in serum-free DMEM medium with various concentrations of H and mH for 1 h, then stimulated with LPS for the indicated times. The cell viability was evaluated using a CCK-8 assay kit (Dojindo, Kumamoto, Japan). All experiments were carried out using 1 μg/mL of LPS for the induction of inflammation.

### 4.4. Measurement of NO Production

The cells were treated with BCS for 1 h, then induced by stimulating them with LPS for 24 h. The culture media was mixed with an equal amount of Griess reagent, reacted at RT for 15 min, and measured at 550 nm using a microplate reader (BioTek Instruments, Inc., Winooski, VT, USA). Serum-free culture medium was used as the blank in all experiments. The amount of nitrite in the samples was obtained by means of the NaNO_2_ serial dilution standard curve and the nitrite production was measured. Relative NO production (%) was calculated as 100 × (nitrite concentration of LPS + sample-treated nitrite concentration of control)/(nitrite concentration of LPS-treated − nitrite concentration of control).

### 4.5. Western Blot Analysis

The protein expression of COX-2, iNOS, MAPK, NF-κB was measured by Western blot analysis. The cells were pretreated with various concentrations of BCS and stimulated with LPS for the indicated times, and the whole cell lysates were extracted with a protein extraction kit (InTRON Biotechnology, Daejeon, Korea). Equal amounts of protein were separated by 10% SDS-PAGE and transferred to PVDF membranes. The membrane was blocked with 5% skim milk in plain buffer (20 mM Tris (pH 7.4), and 136 mM NaCl) at RT for 1 h, then incubated with primary antibodies overnight at 4 °C. Then, the membrane was incubated with 500-times diluted specific secondary HRP-conjugated antibodies at RT for 1 h, and the immunoreactive bands were exposed to enhanced chemiluminescence Western blot analysis detection reagents (ThermoFisher Scientific, Waltham, MA, USA), then were analyzed using a Bio imaging-system (MicroChemi 4.2 Chemilumineszenz-System, Neve Yamin, Israel).

### 4.6. Measurement of Cytokine Production

The levels of proinflammatory cytokines, such as IL-1β, IL-6, TNF-α, and IL-8, in the culture media produced RAW 264.7 cells and HT-29 cells, which were measured by enzyme linked-immunosorbent assay (ELISA) kits (BD OptEIA^TM^, San Diego, CA, USA).

### 4.7. Statistical Analysis

Data are expressed as means ± standard deviation (SD) of at least three independent experiments. One-way ANOVA was used for comparisons of multiple group means followed by the Student′s *t*-test, and statistical significance was considered at *p* < 0.05.
